# Specialism in Medicine—Particularly as Related to Surgery and Gynecology1Read at the fifth annual meeting of the Southern Surgical and Gynecological Association, in Louisville, November 16, 1892.

**Published:** 1892-12

**Authors:** William Warren Potter

**Affiliations:** Buffalo, N. Y.


### SPECIALISM IN MEDICINE—PARTICULARLY AS RELATED TO SURGERY AND GYNECOLOGY.[1]

By WILLIAM WARREN POTTER, M. D., Buffalo, N. Y.

[*Abstract.*]

The time has passed when we may, with propriety, discuss the needfulness of specialism and specialists, because they have grown to be a necessity, and it would not be possible to return to the old way. No one man can cover the whole professional field in teach-

1. Read at the fifth annual meeting of the Southern Surgical and Gynecological Association, in Louisville, November 16, 1892.

ing or practice, because it is already too large, and is constantly widening. If specialists, then, are a necessity, the next question to be considered is the method of preparation for the practice of a specialty.

It is observed that specialists sometimes enter upon practice without adequate preliminary instruction for the work ; and again, they have gone out of the medical schools and immediately begun practice in special lines. Either of these roads to specialism are open to condemnation. Specialists should be properly trained by four years of school life, and then spend six or eight years more in private practice, post-graduate study, and foreign travel. Hence, it would appear that fourteen years is about the average time from entry upon the study of medicine to properly develop a specialist. If specialists are thus trained, they will less frequently fail to recognize the fact that a local fault is oftentimes only an expression of a general dyscrasia, and that the general system frequently needs attention quite as much as the local manifestation of the malady.

The relation of specialists to general practitioners is an important subject for consideration. If there has sprung up an apparent antagonism between them, the specialists themselves must put it down. It is well to recognize the fact that specialists need help from the general practitioner quite as often as the general practitioner needs help from them ; and it will be better for all concerned, and especially for patients, when physicians act on this principle.

A specialist must abandon general practice when he enters upon his chosen field of ministration. The reciprocal relations between specialists is also a matter important to consider, and care must be taken that each department confines itself strictly to its own work and does not encroach upon the boundaries of territory properly belonging to others.

The ethics of specialism may be formulated very simply, and do not require a decalogue of dogmas for its exposition. In 1840, Samuel Jackson, in addressing a class at the University of Pennsylvania, said : "Every man of good sense, possessed of honorable sentiments, and a moral feeling of right and wrong, by the instinct of honesty will know how to conduct himself without a code to regulate his deeds." This is about the sum and substance of all ethics, and is adequate for the government of specialists as well as general practitioners.

The responsibilities of the specialist are now greater than at any other time in our history. The improved methods of teaching in the schools; the massive excellence of medical literature; increased clinical opportunities, and separate State medical examing and licensing boards, all contribute to improve the quality of physicians, and this must apply with as much force to specialists as to others. Schools, however, should be restrained in their tendency to encourage specialism. Their whole energy should be addressed to the teaching and equipment of men for general practice. Specialists will evolve fast enough from the general practitioners so sent out, and this should be the only road to the practice of a specialty.

If general practitioners have become disturbed by the circumstance that tramps have sometimes taken possession of a portion of their territory, they may be consoled with the fact that there yet remains enough to cultivate, and that specialists will be only too glad to assist in driving out these intruders. The argument, then, is:

*First.*—There is essential need for specialists. Divisions of labor in every field are demanded, and nowhere more than in medicine.

*Second.*—Specialists being a necessity, they must equip themselves by years of study, and devote themselves to a still greater number of years of general practice before they are justified in offering themselves as specialists.

*Third.*—They must conduct themselves in such a way as to merit the respect of the general practitioner, and to invite his coöperation in their work.

*Fourth.*—The unwritten ethics of specialism demand that reciprocal relationships be maintained, not only among specialists themselves, but also between specialists and general practitioners.

*Fifth.*—The opportunities for perfection in special lines of medical study are so great, and medical literature, in both journalistic and text-book form, is so rich, that weighty responsibilities are entailed upon the specialist, which must be discharged with fidelity and honor.

*Sixth.*—The schools ought to discourage any and all students who give promise of entering upon the practice of a specialty as soon as the college doors are passed, and before the swaddling clothes of the professional tyro are slipped.

*Seventh.*—The kinship between surgery and gynecology—between the general surgeon and the gynecological surgeon—ought to be strongly encouraged and maintained. Neither should trench upon the territory naturally belonging to the other, and to conserve these reciprocal relations ought to be the pride of this Association.

